# A Case of Imported Leptospirosis: Rhabdomyolysis and Severe Hyperbilirubinemia in a Traveler Returning From Puerto Rico

**DOI:** 10.7759/cureus.34690

**Published:** 2023-02-06

**Authors:** Marcos Garcia, K V Gopalakrishna

**Affiliations:** 1 Internal Medicine, Cleveland Clinic Fairview Hospital, Cleveland, USA

**Keywords:** acute kidney injury, case report, jaundice, rhabdomyolysis, weil’s syndrome, leptospirosis

## Abstract

Leptospirosis is a zoonosis transmitted through human contact with the urine or fecal material of infected animals. Here, we report the case of a young male who presented with hyperbilirubinemia and rhabdomyolysis after returning from Puerto Rico which was confirmed to be severe leptospirosis. An 18-year-old Caucasian male was admitted due to a four-day history of jaundice, fever, headache, abdominal pain, vomiting, dark urine, and pain in his calves. Two weeks before, in Puerto Rico, he swam in caves and at the shoreline in an area recently impacted by a hurricane. Laboratory studies demonstrated leukocytosis, thrombocytopenia, hypokalemia, acute kidney injury with elevated creatine kinase, and hyperbilirubinemia. Due to clinical suspicion of leptospirosis, a serological test was ordered which was positive for *Leptospira *IgM. In this case, the history of swimming in caves and on the shoreline a few weeks after a hurricane that caused flooding in the region made leptospirosis the most likely diagnosis. The patient’s condition improved after initiation of intravenous penicillin G, 8 million units/day, with a resolution of symptoms after completing a seven-day course of antibiotics. Bilirubin started to trend down on day seven, and the patient was discharged on day eight of hospitalization with minimal jaundice. It is important to obtain a detailed medical history when treating patients who have returned from tropical areas, as leptospirosis can mimic other diseases and can be easily mistaken or underrecognized in non-endemic regions, such as the continental United States.

## Introduction

Leptospirosis is a zoonosis transmitted through human contact with the urine or fecal material of infected animals, directly or through exposure to contaminated water or soil. Mammals are the reservoirs and rodents are the primary source of leptospirosis in humans. The causative agent is the bacteria *Leptospira interrogans*, a motile spirochete. It is most commonly seen in tropical climates and usually involves occupational exposure, recreational activities, and travel [1-3].

Several leptospirosis outbreaks have been reported in North America since the 1990s. Most of these outbreaks were associated with flooding or increased rainfall [4]. In the United States, only a few cases are reported every year, and Puerto Rico reports the majority of leptospirosis cases [5]. Leptospirosis outbreaks are especially common during the hurricane season in PR [6,7]. The Centers for Disease Control and Prevention (CDC) data suggest that up to 90% of fatal leptospirosis cases are not reported in Puerto Rico, reflecting underrecognition or underreporting of cases on the island [8,9].

The clinical signs and symptoms of human leptospirosis vary and can resemble several other common diseases. Leptospirosis shares many clinical signs and symptoms with other febrile illnesses, particularly those that are prevalent in tropical regions, including dengue, zika, yellow fever, viral hepatitis, rickettsia infection, malaria, hantavirus, typhoid fever, HIV infection, and bacterial sepsis [10].

The level of suspicion for leptospirosis in a given patient depends primarily on the clinical syndrome and the local epidemiology for this infection. Therefore, we report a unique case of severe leptospirosis in a previously healthy young man who acquired the infection while traveling in Puerto Rico but became ill only after returning to Ohio.

## Case presentation

An 18-year-old Caucasian male from the continental United States, with no past medical history, presented to an emergency department (ED) in Ohio with a four-day history of jaundice, fever, chills, headache, abdominal pain, nausea, vomiting, non-bloody diarrhea, dark urine, weakness, and diffuse myalgias with pain in his calves.

A recent travel to Puerto Rico was mentioned by the patient 14 days prior to admission. He had visited a community in Ponce, in the southern part of the island, which is an area that had been impacted by Hurricane Fiona six weeks prior to his travel. He claimed to have gone swimming in local caves and at the beach. He only consumed bottled water and denied consuming raw foods or being around ill people in Puerto Rico; however, he described getting bitten by insects. He denied drinking, smoking, or using recreational drugs. There were no recent vaccinations or interactions with animals.

Upon admission, the patient’s vital signs were significant for tachycardia (120 beats per minute), fever (101.5°F), and tachypnea (24 breaths per minute). He also demonstrated pulse oximetry of 97% on room air and a body mass index of 18 kg/m^2^. In addition, he was icteric, with conjunctival injection but no ocular suffusion. He had normal breath sounds bilaterally with no murmurs, and his abdomen was soft but tender to palpation in the epigastrium.

Laboratory studies demonstrated leukocytosis, with neutrophil predominance, thrombocytopenia, anemia, hyponatremia, hypokalemia, acute kidney injury (AKI), and increased creatinine levels on admission (baseline was 0.7 mg/dL). Creatine kinase, alanine aminotransferase (ALT), aspartate aminotransferase (AST), and bilirubin were significantly elevated. Ammonia levels and coagulation studies were within normal limits. Urinalysis revealed large amounts of blood and bilirubin. The rapid HIV test was negative. Laboratory tests are described in Table 1.

**Table 1:** Laboratory data during the hospital stay. INR: international normalized ratio; IgM: immunoglobulin M

Laboratory test	Day one	Day two	Day three	Day four	Day eight	Reference range
White blood cell count	13.85	9.39	12.95	15.40	13.82	3.7–11.0 k/µL
Hemoglobin	11.7	9.3	9.7	9.9	11.1	13–17 g/dL
Platelets	44	64	97	127	248	150–400 k/µL
Sodium	133	141	140	137	141	136–144 mmol/L
Potassium	3.4	3.3	4.3	4.0	4.2	3.7–5.1 mmol/L
Magnesium	1.9	1.3	1.5	1.4	1.4	1.7–2.3 mmol/L
Blood urea nitrogen	46	26	15	12	18	9–24 mg/dL
Creatinine	2.41	1.31	0.85	0.65	0.77	0.73–1.22 mg/dL
Albumin	3.6	2.5	2.9	3.1	3.5	3.9–4.9 g/dL
Total bilirubin	21.2	29.3	40.6	43.1	8.9	0.2–1.3 mg/dL
Conjugated bilirubin	18.7	>20.0	>20.0	>20.0	7.1	<0.2 mg/dL
Alkaline phosphatase	119	92	127	132	136	55–149 U/L
Aspartate transaminase	358	414	364	246	98	14–40 U/L
Alanine transaminase	112	128	153	152	154	10–54 U/L
Creatine kinase	12,692	15,330	8,886	2,706	174	51–298 U/L
Myoglobin	1,686		298	99	75	<91 ng/mL
Prothrombin time (INR)	1.0	1.0	0.9	1.1	1.0	0.9–1.3
Leptospira IgM			Positive			Negative

The patient was admitted to a regular medical floor, with suspicion of infection or inflammation in the hepatobiliary system. Computerized tomography of the abdomen showed only mild mesenteric edema and free fluid in the pelvis. The right upper quadrant ultrasound was unremarkable. Differential diagnoses included cholangitis, acute pancreatitis, hepatitis (viral, autoimmune, drug- or toxin-associated), leptospirosis, dengue fever, typhoid fever, histoplasmosis, infectious colitis, and salmonellosis.

Ceftriaxone was started due to a strong clinical suspicion of leptospirosis. In the hospital, he continued to demonstrate nausea, but his diarrhea resolved on the day of admission. On the following day after admission, due to increased bilirubin and suspected risk of ceftriaxone-induced hyperbilirubinemia, ceftriaxone was discontinued and he was started on penicillin G. Due to local availability, only a serological test was ordered on the day of the admission, which was significantly positive for *Leptospira *IgM by dot blot enzyme-linked immunosorbent assay. Hepatitis panel and serologies for Epstein-Barr virus, cytomegalovirus, dengue fever, and histoplasmosis were negative.

After the initiation of intravenous (IV) penicillin G, the patient’s condition improved dramatically, with the resolution of nausea, fever, and abdominal pain on the first two days of the admission. On day three of hospitalization, his kidney function was back to normal, and creatine kinase and myoglobin were trending down rapidly. However, his bilirubin continued to trend up until day five (peak total bilirubin 48 mg/dL). The bilirubin started to trend down only on day seven, and the patient was discharged on day eight of hospitalization with minimal jaundice. The patient completed a seven-day course of IV penicillin G, 8 million units/day, in divided doses every six hours and was discharged after demonstrating clinical improvement. Upon discharge, the patient demonstrated the ability to maintain a standard dietary regimen. A follow-up appointment was arranged with the infectious disease department in two weeks, however, the patient failed to attend.

## Discussion

From 1945 to 1994, up to 150 cases of leptospirosis were reported annually in the United States; however, after 1994, the disease was no longer designated as a nationally notifiable condition [11]. Leptospirosis was reinstated as a nationally notifiable disease in 2013, and, since then, it is estimated that 100-200 leptospirosis cases are identified annually in the United States, most of them in Puerto Rico or Hawaii [12].

Leptospirosis can present with a wide range of symptoms in humans, ranging from a mild, self-limiting fever to a more severe, potentially fatal illness that affects multiple organs [13]. Severe cases of leptospirosis are characterized by dysfunction in various organs such as the liver, lungs, brain, and kidneys. A specific syndrome that can occur in severe cases is known as Weil’s disease, which is characterized by jaundice, liver injury, and kidney injury and can occur in up to 10% of patients with a high mortality rate [10,13].

The incubation period is usually 5-14 days, with a range of 2-30 days [5]. In this case, the patient’s symptoms started on the 10th day after returning from his travel, which is within the described range for incubation.

In patients with a severe form of leptospirosis, damage to the cells of the liver and the breakdown of connections between liver cells can cause bilirubin to leak out of the bile [13]. There is a disproportionate increase in conjugated bilirubin accompanied by mild-to-moderate elevations in transaminases which rarely exceed 200 IU/L because hepatic necrosis is rare in such patients. Importantly, bilirubin levels can take more than five weeks to fully normalize [14].

Our patient exhibited a unique clinical manifestation of severe leptospirosis, characterized by the presence of jaundice, AKI, and elevated bilirubin levels, without the typical signs of bleeding or respiratory failure. Interestingly, the patient developed rhabdomyolysis. It is noteworthy to mention that it took three days for a 50% decrease in the direct bilirubin levels from its peak.

Initial kidney involvement in leptospirosis is characterized by a unique non-oliguric hypokalemic renal injury [15]. Tubular function alterations in leptospirosis precede the fall of the glomerular filtration rate, explaining the early findings of hypokalemia and hypomagnesemia [16]. Renal vasoconstriction, tubular obstruction, and direct myoglobin toxicity are the main mechanisms of renal failure after rhabdomyolysis [17]. High creatine kinase levels were found more frequently in patients with severe AKI than in those with mild AKI, suggesting that rhabdomyolysis itself may contribute to the severity of AKI [18].

Leptospirosis is typically diagnosed through serological methods due to the limitations of culture and polymerase chain reaction (PCR). The presence of IgM antibodies for *Leptospira *in the blood can be detected within five to seven days of the onset of symptoms, making it a more sensitive method of diagnosis in the early stages of the illness compared to the microagglutination test (MAT). In this particular case, the diagnosis was hindered by logistical challenges, and it was not possible to perform PCR or MAT testing. Additionally, cultures were not conducted due to a lack of access to specialized media.

According to the CDC, prompt initiation of treatment has been shown to lead to a milder course of the disease with a shorter duration of symptoms. When there is a strong clinical suspicion of leptospirosis, it is advisable to start antibiotics without delay. In cases of mild leptospirosis, doxycycline is the preferred drug of choice. In severe cases of leptospirosis, IV penicillin is the recommended treatment option, with ceftriaxone being equally effective [5].

## Conclusions

Our patient’s travel history and clinical presentation strongly suggested a severe case of leptospirosis, particularly when considering the details of his trip to Puerto Rico and the recent flooding caused by Hurricane Fiona in the area. The presence of fever, jaundice, calf pain, hypokalemic renal failure, and rhabdomyolysis are key indicators of severe leptospirosis, even in the absence of signs of bleeding, ocular suffusions, or pulmonary infiltrates. It is crucial to take a detailed medical history when treating patients who have returned from tropical areas, as leptospirosis can mimic other diseases and may be easily mistaken or underrecognized in non-endemic regions such as the continental United States. The appropriate antibiotic therapy should not be delayed while waiting for diagnostic test results.
